# Has China’s hierarchical medical system improved doctor-patient relationships?

**DOI:** 10.1186/s13561-024-00520-8

**Published:** 2024-07-18

**Authors:** Yang Gao, Yang Yang, Shoupeng Wang, Wenqian Zhang, Jiao Lu

**Affiliations:** 1 School of Economics and Management, Northwest University, Xi’an, Shaanxi China; 2 School of Economics, Qufu Normal University, Rizhao, Shandong China; 3 School of Public Policy and Administration, Xi’an Jiaotong University, Xianning West Road 28#, Xi’an, 710049 Shaanxi China

**Keywords:** Healthy China, Hierarchical Medical System, Doctor-Patient relationships, Resource misallocation, Difference-in-Differences Analysis

## Abstract

**Background and objective:**

Developing harmonious doctor-patient relationships is a powerful way to promote the construction of a new pattern of medical reform in developing countries. We aim to analyze the effects of China’s hierarchical medical system on doctor-patient relationships, thus contributing to China’s medical and health system reform.

**Methods:**

With panel data on prefectural-level cities in China from 2012 to 2019, we used a time-varying difference-in-differences model to evaluate the effect of hierarchical medical treatment policy.

**Results:**

Hierarchical medical treatment policies can significantly improve doctor-patient relationships, and this conclusion is supported by various robustness tests. And improving doctor-patient relationships can be indirectly realized by the optimization of resource allocation and saving of medical costs. In addition, the marginal effect of the pilot policy on doctor-patient relationships decreased with age within the city population. In focal cities and cities with high levels of fiscal spending on health care, the effect of the pilot policy on doctor-patient relationships was stronger.

**Conclusion:**

While reinforcing the literature on the doctor-patient relationship, this study also provides a reference for further exploration of the pilot policy of hierarchical medical treatment and the development of new medical and health system reform in developing countries.

## Introduction

Harmonious society is a kind of good society that mankind strives for, a social state of harmony, togetherness and concerted efforts at all levels. However, as the medical and healthcare system reform of China has entered the “deep water zone” and “period of attack”, the intricate relationship of interest has led to frequent medical accidents, overburdened liability compensation for medical damage, and “medical trouble” incidents, and conflicts between doctors and patients are constantly escalating [1, 2]. According to the 2018 White Paper on the Practice Status of Chinese Physicians, approximately 62% of physicians have experienced medical disputes of varying degrees. According to the 2020 National Big Data Report on Healthcare Damage Liability Dispute Cases, the total number of healthcare damage liability dispute cases in China was 18,670, up about 3% from 2019. The frequent occurrence of medical disputes has become a “bottleneck” that restricts the process of medical and healthcare system reform and the construction of a harmonious society. Promoting harmonious doctor-patient relationships has become a new era of building a healthy China, and harmonious social development urgently needs to compensate for these shortcomings and weaknesses.

The doctor-patient relationship refers to the interactive relationship between doctors and patients, as well as social groups and individuals whose interests are closely related to those of both parties, which is formed in medical services [3, 4]. The doctor-patient relationship is not only the basis for ensuring the smooth implementation of medical activities and is closely related to the immediate interests of both doctors and patients but also an essential part of building a harmonious society. Compared with countries such as North Korea, Russia, and Sweden, which have adopted a “universal free medical system”, doctor-patient conflict is very prominent in China [5, 6]. Although China has carried out numerous medical and healthcare system reforms, which have had an impact on the quality of healthcare services, the fundamentally tense doctor-patient relationship remains largely unchanged, leading to patient suffering, healthcare grievances, and uneasiness.

To address doctor-patient conflicts in depth, most of the literature has studied doctor-patient relationships at the micro level and revealed that burnout, lack of knowledge of health information, and poor communication between doctors and patients can exacerbate the asymmetry of information between doctors and patients and lead to further deterioration of doctor-patient relationships [7, 8]. However, the macrolevel factors hidden in the system are the key to the doctor-patient relationship. The root cause of the deterioration of the doctor-patient relationship is the problem of “difficult and expensive access to medical care”, and the core cause of the problem of “difficult and expensive access to medical care” is the irrational distribution of medical resources [9]. Chan [10] noted that at present, the utilization pattern of medical services in the Chinese healthcare sector presents an “inverted pyramid” type, and the structural imbalance of “aggregation at the top” has led to the problem of “difficult and expensive seeing a doctor” becoming increasingly prominent. On the one hand, patients take the initiative to “seek upward medical treatment” out of their preference for high-quality medical resources, resulting in large-scale hospitals being crowded with patients, overutilizing resources, and increasing the cost of medical congestion; on the other hand, primary healthcare institutions are deserted, and a large amount of medical equipment and human resources are left unused. The “inverted pyramid” structure reflects the irrational distribution of healthcare resources in China, where the tension of healthcare resources in large hospitals coexists with the waste of healthcare resources in primary hospitals, leading to the problem of “difficultly seeing a doctor and expensive seeing a doctor”, which has become a core pain point affecting the harmonious development of doctor-patient relationships [11, 12].

The government, as a “tangible hand”, can reduce the irrational allocation of resources, which in turn helps to solve the chronic problem of “difficultly seeing a doctor, expensive seeing a doctor” and improve the doctor-patient relationship. In September 2015, the State Council issued the “General Office of the State Council on the Promotion of Graded Diagnosis and Treatment System Guidelines on Promoting the Construction of Graded Diagnosis and Treatment System. The hierarchical medical system is clearly an important measure for the rational allocation of medical resources and the promotion of the equalization of basic medical and health services. The hierarchical medical treatment policy refers to the gradation of illnesses according to their severity and degree of difficulty, with medical institutions at different levels undertaking the treatment of different illnesses and gradually realizing the process of medical treatment from general practice to specialization. The basic connotation of the hierarchical medical treatment policy is primary care, two-way referral, emergency and slow treatment, and up-and-down linkages. Through continuous exploration, five models have been developed: family doctor contract services, medical insurance policy guidance, medical associations, chronic disease management and diagnosis and treatment. The hierarchical medical treatment policy is not only an important part of China’s healthcare system reform but also a bold attempt by the government to benefit more people with minimal investment. By 2020, the pilot policy of hierarchical medical treatment had covered 31 provinces (autonomous regions and municipalities directly under the central government) in China.

Theoretically, the hierarchical medical treatment policy can optimize the allocation of resources and save medical resources, “difficultly seeing the doctor, see the doctor expensive” this disease to carry out effective “targeted treatment”, thus promoting the construction of a harmonious doctor-patient relationship. On the one hand, the hierarchical medical treatment pilot policy has the advantages of optimization the source allocation of different levels of healthcare institutions, improving the efficiency of the utilization of medical resources, and improving the order of medical treatment, which will enable grassroots healthcare institutions to give full play to the “bottom of the network” role in healthcare institutions [13, 14]. On the other hand, hierarchical medical treatment policies can save the cost of medical care for residents by providing accessible, comprehensive, continuous, and coordinated medical services to the population [15]. Although the literature suggests theoretically that the hierarchical medical treatment pilot policy can improve the doctor-patient relationship, solid empirical evidence is needed for effective testing. Therefore, this paper attempts to answer the following questions: does the hierarchical medical treatment pilot policy improve the doctor-patient relationship? If so, what is its impact path? Is there heterogeneity in improvement effects?

Therefore, this paper takes the hierarchical medical treatment pilot policy implemented by different municipalities in different years as exogenous shocks and constructs a time-varying difference-in-differences model based on municipal panel data from 2012 to 2019 to analyze in detail the medical relationship improvement effects of the hierarchical medical treatment pilot policy. The study revealed that the hierarchical medical treatment pilot policy can improve the doctor-patient relationship, a conclusion that still holds after considering a series of robustness tests. Mechanistic tests revealed that the optimization of resource allocation and saving of medical costs were significant pathways through which the hierarchical medical treatment pilot policy improved the doctor-patient relationship. Heterogeneity analysis revealed that the marginal effect of the hierarchical medical treatment policy on improving doctor-patient relationships diminishes as the degree of aging increases in each city and that the hierarchical medical treatment pilot policy is more effective at improving doctor-patient relationships in key cities and cities with high financial health care expenditures.

To summarize, this paper tries to be innovative in the following aspects on the basis of previous studies. First, from the perspective of doctor-patient conflict, this paper attempts to reveal the impact of hierarchical medical treatment policy on the doctor-patient relationship, not only to expand the role of hierarchical medical treatment policy boundaries but also to expand and supplement the literature related to the relationship between doctors and patients. Second, this paper explores how hierarchical medical treatment policy affects doctor-patient relationships by examining the two paths of resource allocation optimization and medical cost savings, which not only unravels the mystery of the mechanism of the path of hierarchical medical treatment policy but also provides specific paths to improve the doctor-patient relationships. Furthermore, this paper further explores the heterogeneous impact of hierarchical medical treatment policy from three perspectives—the degree of urban aging, urban class, and urban financial health care expenditures—which not only deepens the understanding of the intrinsic law of hierarchical medical treatment policy affecting the doctor-patient relationship but also provides a direction for improving the effectiveness of hierarchical medical treatment policy in a targeted manner. Finally, this study helps to clarify the implementation effects and potential problems of the current pilot hierarchical medical treatment policy from the perspective of improving the doctor-patient relationship, thus providing important empirical evidence for realizing the overall goal of the “Healthy China “2030” Planning Outline. It also provides methods and references for the reform of China’s healthcare system in nonpilot areas and provides Chinese solutions and wisdom for other developing countries to build a new pattern of healthcare reform.

The remaining parts of this article are arranged as follows: The second section includes the literature review. The third section covers the institutional background and theoretical analysis. The fourth part describes the research design, including the model setting and the variable definitions, sample selection and data sources. The fifth, sixth, and seventh parts include the empirical analysis, including the baseline regression, the common trends test and dynamic effect analysis, robustness tests, solutions to endogeneity, and the mechanism and heterogeneity analyses. The last section discusses the conclusion and policy implications.

## Literature review

To address this research theme, this paper reviews and comments on the following three types of literature: related studies on social harmony, the measurement and factors influencing the doctor-patient relationship, and the empowering effects of healthcare medical policy.

### Related studies on harmonious society

The harmonious society has always been a common pursuit of mankind. As early as 1893, Durkheim proposed the theory of social integration and emphasized that intergroup conflicts, transgressions, extreme individualism, and misbehavior are all threats to building a harmonious society. Furthermore, Rawls (1971) noted that promoting social equity contributes to social cohesion and social harmony. On this basis, Chinese scholars have also expanded the connotation of social harmony. For example, Lu and Liu [16] argued that social harmony is the internal harmony between different fields of society, between different industries, and between subjects with diversified interests, as well as between each other, and even the ultimate harmony of society as a whole. Wang and Xiao [17] noted that the core goal of the construction of a harmonious society is to promote relationships between all aspects of society to show harmony and coordination and all kinds of interests to be effectively solved and addressed and ultimately to promote the effective realization and maintenance of social justice. However, in recent years, due to the inconsistency of interests between doctors and patients, the two sides do not trust each other, which in turn leads to the frequent occurrence of violent injuries to doctors, exacerbating the contradiction between doctors and patients and worsening the doctor-patient relationship [18]. The deterioration of doctor-patient relationships will not only directly affect the quality of medical activities and jeopardize the immediate interests of both doctors and patients but also further exacerbate the contradiction between supply and demand in the field of health care, seriously hindering the construction of a harmonious society.

### Measurements related to doctor-patient relationships

Most of the literature has investigated doctor-patient relationships using questionnaires. For example, Deng et al. [5] utilized data from 908 questionnaires from the Fifth National Health Services Survey in China in 2013 to reflect the doctor-patient relationship through the scores of four questions: patients are very respectful to me, my profession is respected in society, patients trust me, and the current doctor-patient relationship is very good. Li et al. [18] used the doctor-patient relationship questionnaire that was developed on the basis of the PDRQ-9, which assesses nine dimensions, such as helpfulness, adequacy of time, trustworthiness, understanding, dedication, consistency, and accessibility to the doctor, patient satisfaction with care, and patient satisfaction with care. Higher scores represent better doctor-patient relationships. However, the use of questionnaires, on the one hand, can only survey a portion of the public and cannot cover the entire population, and the conclusions drawn may be biased; on the other hand, the questionnaire survey may be subject to the impact of the subjective emotions and moods of the respondents at that time. Therefore, some scholars have attempted to objectively reflect doctor-patient relationships through the Baidu index and litigation records. For example, Liu et al. [19] used the Dagum Gini coefficient, kernel density estimation, and spatial autocorrelation to characterize the spatial and temporal differences in concerns for doctor-patient relationships based on the Baidu index of provincial doctor-patient disputes from 2011 to 2019 and found that concern for doctor-patient relationships in China has increased overall and spatially presented an unbalanced pattern of decreasing and becoming increasingly obvious from east to west [20]. Xiao et al. [12] analyzed the data of litigation records of violence against health personnel in China’s adjudication network from 2013 to 2021 and found that doctor-patient relationships in China have become increasingly tense [2].

### Influences on the doctor-patient relationship

A good doctor-patient relationship is fundamental to ensure the normal conduct of the medical process [21]. The influencing factors of the doctor-patient relationships should be explored so that the right medicine can be targeted from the source to improve the doctor-patient relationship. At the micro level, doctor satisfaction and patient satisfaction are the key factors affecting the doctor-patient relationships. From the doctor’s perspective, the doctor’s level of diagnosis and treatment, level of communication skills, and responsibility are closely related to the caliber of diagnosis or treatment received by the patient [22], which profoundly affects the patient’s experience visiting the clinic and thus the doctor-patient relationships. Moreover, Ahmad et al. [23] noted that physician burnout severely reduces the quality of healthcare services, and poorer-quality healthcare services are significantly associated with the deterioration of doctor-patient relationships [24]. From the patient perspective, on the one hand, a lack of patient knowledge of health information exacerbates the degree of doctor-patient information asymmetry, triggering difficulties in doctor-patient communication and leading to the deterioration of doctor-patient relationships [25]. On the other hand, patients’ unrealistic demands and expectations exacerbate the degree of tension in doctor-patient relationships [8]. For this reason, Jiang et al. [2] emphasized that changing patients’ inappropriate behaviors and philosophies is a key initiative to de-escalate the patient-physician relationship, which in turn requires that physicians be competent enough and have sufficient clinical time to modify patients’ perceptions of health [26]. In addition, Zhou et al. [27], Xu [21] and Wang et al. [1] argued that online social media (e.g., Facebook, Twitter, etc.) or online health wellness information also influences the doctor-patient relationship.

The deterioration of the doctor-patient relationship involves numerous factors, ranging from the triggering of specific events at the micro level to deep-seated macrolevel problems. At the macro level, the “top aggregation” of medical resources, patients due to insufficient knowledge of the condition, coupled with the uneven distribution of resources between urban and rural areas and regions, makes residents “upward to seek medical treatment”; thus, the phenomenon of “difficultly finding a doctor” frequently occurs, the pressure on doctors to receive treatment is too great, and the difference between the patient’s waiting time and the time of receiving treatment is too great, which leads to tensions in the doctor-patient relationships. For primary medical institutions, due to the relative insufficiency of medical resources and the backwardness of medical technology, coupled with the stereotypical impressions of patients, a large amount of medical equipment and human resources have been left unused. The disordered nature of the medical process causes the phenomenon of “difficult and expensive seeing a doctor” becoming increasingly serious, exacerbating the negative emotions of patients, such as tension, anxiety, and irritability, which makes the doctor-patient relationship constantly tense. As Deng et al. [5] noted, one of the fundamental reasons for the greatly increased incidence and severity of medical disputes is that health care services are not effectively divided into hospital health care and primary health care. In other words, rational allocation of healthcare resources is a key means to reshape harmonious doctor-patient relationships.

In addition, a poor doctor-patient relationship is not only a one-way process but also a product of a vicious cycle. Doctors’ tension toward patients may affect their diagnostic and therapeutic decisions, leading to defensive diagnosis and overmedication to safeguard their own rights and interests. This overmedication not only increases the burden of patients’ medical care but also reduces patients’ trust and expectations of doctors, leading to a decrease in patient satisfaction, which in turn affects the effectiveness of diagnosis and treatment and exacerbates doctor-patient conflict. Moreover, Lu [28] noted that poor doctor-patient relationships also negatively affect the number of medical students, further exacerbating the imbalance between the supply and demand of medical resources. Solving the problem of strained doctor-patient relationships has become an urgent task.

### The empowering effects of healthcare medical policy

The pilot policy of hierarchical medical treatment, as a kind of macromedical policy whose policy goal is directly directed to the reshaping of the order of medical consultation, is of great significance for the construction of a harmonious doctor-patient relationship and may become the optimal decision to alleviate the conflict between doctors and patients [29, 30]. Yang and Fu [31], taking Chongqing as an example, found that the implementation of a pilot policy of hierarchical medical treatment significantly reduced the average medical burden of urban residents. Zhang and Chen [32], taking Xiamen as an example, confirmed that the pilot policy of hierarchical medical treatment effectively guided patients with chronic diseases to sink to primary health care institutions for treatment. Wang and Sun [33] collected 1165 valid patient questionnaires in Shanghai, used descriptive statistics and Ordered Logistic Regression Model (OLM) for empirical analysis and found that the satisfaction and loyalty of the interviewed patients to primary hospitals increased after the reform of hierarchical medical treatment. Based on the data of the 2012–2018 China Family Panel Studies (CFPS), Zhou et al. [34] found that the implementation of the pilot policy of hierarchical medical treatment effectively changed the original disorderly medical behavior of residents and led to the reshaping of the order of medical consultation [35].

Although existing studies have not analyzed the impact of hierarchical medical treatment policy on doctor-patient relationships at this time, the impact of other health care policies on doctor-patient relationships in existing studies can provide a reference for this paper. For example, by analyzing the association between health policies and doctor-patient relationships in China from 1949 to 2015, Zhou et al. [36] found that hierarchical medical treatment policy is the main factor determining the quantity and quality of public health services and is a key variable affecting doctor-patient relationships. Unreasonable health care policies can exacerbate the opposition of interests between doctors and patients and deteriorate doctor-patient relationships. Yan et al. [37] found adopting a global budget scheme to carry out a cost-control policy that fixes the amount of the budget, allocates funds, and provides financial regulation can have an impact on physician and patient satisfaction in a variety of ways, which in turn affects the physician-patient relationship through a questionnaire survey of 110 hospital clinicians. Gu et al. [7], based on data from three regions, Jiangsu, Shandong and Hubei provinces, found that contractual service policies such as contracting with rural physicians and outsourcing medical services had a positive effect on establishing a harmonious doctor-patient relationship, and physician communication skills were a mediator of the influence of contractual service policy on the doctor-patient relationship. Li et al. [18] conducted a cross-sectional survey of 16 patients with chronic diseases in 847 primary care organizations in Jilin Province and found that contractual services could directly or indirectly influence patients’ perceived engagement, which in turn influenced the relationship between doctors and patients. Gu et al. [38], based on questionnaire data from 574 rural clinics in three counties in China, reported that the implementation of family physician contract services played a key role in changing doctor-patient relationships and helped to attract more patients to primary care centers.

According to the literature, although relevant theoretical and empirical studies have yielded high-quality research results, there are still some problems to be addressed. First, the established literature on the evaluation of doctor-patient relationships is mostly based on self-assessments of the patient’s degree of satisfaction with the patient and other indicators, but the above indicators are strongly subjective, which can lead to the distortion of causal inference. Second, from the perspective of the factors influencing doctor-patient relationships, the current research on the issue of doctor-patient relationships has been carried out mostly at the microlevel, and macrolevel discussion is relatively lacking. Most of the literature on the factors influencing doctor-patient relationships involves theoretical analysis or descriptive statistical analysis based on cross-sectional data, and relatively few empirical studies exist. Finally, although the improvement effect of the hierarchical medical treatment pilot policy on the health care system has been theoretically confirmed, its impact on the doctor-patient relationship and the path of hierarchical medical treatment is still relatively uncharted and urgently needs to be thoroughly explored.

## Institutional background and theoretical analysis

### Institutional context

After the 1880s, China drastically cut government investment in the primary healthcare service system, and primary healthcare institutions rapidly shrunk and experienced aging equipment, outdated technology, and a lack of human resources, which led to a further expansion of the “Matthew effect” in the healthcare service capacity between primary healthcare institutions and high-level hospitals [39, 40]. To realize the ambitious goal of providing fair and high-quality basic medical and health services to the entire population and reducing the economic burden of disease among the masses, China has opened a new round of medical and health system reforms and has taken the reform of the primary medical and health care service system as one of the core elements.

**Fig. 1:**
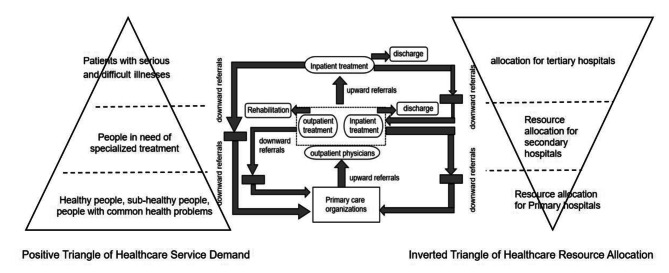
Operation mechanism and resource allocation of China’s hierarchical medical system

The hierarchical medical treatment policy aims to promote the downward focus of medical and health work, the decrease in quality medical resources and the improvement of community-level medical services and has always been the focus of reform of the community-level medical and health service system. In 2009, the Opinions of the CPC Central Committee and The State Council on Deepening the Reform of the Medical and Health System proposed guiding general medical and treatment to the grassroots level and gradually realizing the first diagnosis in the community, hierarchical medical treatment and two-way referral. In 2013, the Decision of the CPC Central Committee on Several Major Issues Concerning Comprehensively Deepening Reform explicitly proposed to improve the rational hierarchical medical treatment model and to establish a contractual service relationship between community doctors and residents, and in 2015, the General Office of the State Council issued the Guiding Opinions on the Advancement of the Construction of a Hierarchical Medical System, which marked the official promotion of the system of hierarchical medical policy and treatment as a core content of the new healthcare reform on a nationwide scale. In the same year, the relevant departments adopted a series of closely related policy initiatives to promote hierarchical medical treatment in conjunction with the system and to guide residents to seek medical care voluntarily and in an orderly manner, including the community health service upgrading project, the comprehensive reform of urban public hospitals, the “Internet+” benefit service, and the pilot work on hierarchical medical treatment of chronic diseases. In 2016, the National Health and Wellness Conference positioned hierarchical medical treatment policy as the first of the five basic medical and healthcare systems. In 2017, the State Council issued the “Thirteenth Five-Year Plan for Deepening the Reform of the Medical and Healthcare System”, which made it clear that a system of hierarchical medical treatment in line with national conditions would basically be established by 2020. In 2018, in the National Health and Wellness Medical Department, in its “Notice on Further Notice on Further Doing the Key Work Related to the Construction of the Hierarchical Medical System”, the NHMH emphasized key initiatives in the planning and construction of medical associations and the improvement of the guarantee policy for the “four separations” of hierarchical medical treatment (separation of regions, urban and rural areas, upper and lower levels, and urgency and slowness). In 2019, the NHMH issued the “Notice on Doing a Good Job of Signing Services for Family Doctors”, further emphasizing that the focus of hierarchical medical treatment is on improving the capacity of primary healthcare services.

hierarchical medical treatment policy centers on the grading of diseases and the grading of medical institutions, which refers to the grading of diseases according to their lightness, severity, slowness and urgency, and requires medical institutions at different levels to treat diseases at different levels, as shown in Fig. 1. Medical institutions in the same region are divided into first-, second-, and third-level medical institutions according to their functions and scope of services. Primary medical institutions, also known as primary medical institutions, are the first level of the hierarchical medical system and are usually used for basic health care services provided by general practitioners, family doctors and community health centers, focusing on providing treatment, rehabilitation and nursing care for patients with chronic illnesses who have a clear diagnosis and stable condition, patients in rehabilitation, elderly patients and patients with advanced tumors, among others. Secondary medical institutions provide specialized medical services by specialists and hospitals in need of more advanced diagnostic and treatment facilities and are mainly used in hierarchical medical treatment to receive patients referred by tertiary medical institutions for recovery from acute illnesses, postoperative recovery, and stabilization of patients with critical illnesses. Tertiary medical institutions are the highest level of medical institutions and involve highly specialized medical services provided by specialist hospitals and medical centers. Tertiary medical institutions are mainly responsible for treating difficult and critically ill patients and carrying out teaching and scientific research in the context of hierarchical treatment while also needing to refer patients whose conditions have improved to general hospitals for routine care.

In concrete practice, hierarchical medical treatment policy matches the demand for medical and health care services with the allocation of medical and health care resources in a model of primary-level first care, two-way referral, emergency and slow treatment, and up-and-down linkage to achieve the goal of “keeping minor illnesses out of the villages, keeping common illnesses out of the towns (townships), keeping major illnesses out of the counties, and referring those with difficult or critical illnesses”. Primary care refers to adhering to the principle of voluntary participation by the public and encouraging patients with common and frequent illnesses to go to primary medical and healthcare institutions first through policy guidance. Two-way referral refers to improving referral procedures, focusing on smooth downward referral of patients in the chronic and recovery phases and gradually realizing orderly referral between different levels and types of medical institutions. Separation of acute and chronic diseases refers to improving the service system for subacute and chronic diseases, transferring patients who have spent the acute stage from tertiary hospitals, and realizing the functions of acute and chronic disease diagnosis and treatment services of all levels and categories of medical institutions. Up-and-down linkage refers to the establishment of a division of labor and collaboration mechanism between medical institutions and the promotion of the vertical flow of high-quality medical resources.

All parts of China are constantly exploring and practicing the construction of hierarchical medical systems and have already accumulated a certain amount of experience. The system enhances the capacity of primary medical services and guides the public to seek primary medical care in an orderly manner by combining medical institutions at different levels, implementing differentiated payments for medical insurance, setting up multidisciplinary teams of medical specialists, and improving the standardization of the diagnosis and treatment of common diseases and the use of medication.

### Theoretical analysis

The hierarchical medical treatment policy can promote the optimization of resource allocation and saving of medical cost through the sinking of medical and health care resources, which can improve the doctor-patient relationship, as discussed below.

#### Optimization of resource allocation

China’s administrative model for allocating resources favors high-level hospitals, which leads to an inflow of talent to these hospitals. Coupled with the purchase of high-quality medical equipment, a comprehensive system for high-level healthcare services has been formed. However, given the current lack of “gatekeepers”, patients choose where to seek medical treatment not based on their actual condition but rather on the basis of the hospital level, which acts as a “signal” of quality. Patients blindly choose higher-level hospitals and book higher-level doctors when seeking healthcare services, resulting in a discrepancy between the actual recipients of medical services from higher-level hospitals and those who actually need such high-level services. As many as 70% of the patients admitted have common diseases, which causes highly skilled doctors to be overworked for extended periods [41]. The workload at higher-level hospitals exceeds their carrying capacity, which greatly increases the waiting time for patients seeking medical treatment, shortens consultations, and occasionally causes unpleasant experiences [42]. Another manifestation of the irrational allocation of medical resources is primary health-care organizations, which have extremely limited medical resources. Weak resource support greatly reduces their attractiveness to medical professionals, creating a situation where “the best is better than the best, and the worst is worse than the worst”, which is prone to misdiagnosis and tragedy. This further reduces patients’ trust, distorts their decision-making, and exacerbates the imbalance in the utilization of medical resources [43]. In the end, due to the long waiting times for medical treatment and the poorer-than-expected results from healthcare services, patient satisfaction with medical treatment is reduced, which leads to dissatisfaction with or indifference toward doctors [1].

As a source of progress in the medical system intended to increase efficiency, the primary purpose of hierarchical medical treatment policy is to rationally allocate medical and health resources and improve the way in which medical treatment is provided [29]. First, on the patient side, the hierarchical medical treatment policy creates a stepwise reimbursement model for medical institutions of different levels. In this model, there is a 5–10% gap in the reimbursement ratio between medical institutions of different levels. This gap is introduced to fully address the ability of medical insurance to guide health-seeking behavior. Punitive measures are implemented for patients who seek treatment out of order and leapfrog over certain level institutions. This approach reshapes the “gatekeeper” system and standardizes the order in which medical treatment is received [14]. Second, on the medical provider side, it is necessary to balance investments in medical resources and to increase investment in the infrastructure of primary care facilities. Increasing subsidies for medical staff at primary care facilities and emphasizing skills training to enhance their ability to diagnose conditions and provide treatment will thereby improve the levels and quality of the medical services available at primary care facilities, making such facilities a reasonable choice for patients seeking medical treatment. Initially, patients were guided to choose the correct medical institution based on their condition, thus alleviating the mismatch in the supply and demand of medical resources at higher-level hospitals and improving patient satisfaction [44].

#### Saving of medical cost

There are structural constraints in the current medical institution management and drug supervision systems, which prevent the effective supervision of doctor behavior. This results in a percentage of the gap between the wholesale and retail prices of medicines being retained as hospital income, which intensifies the excesses in the prescribing behavior of doctors induced by profit-seeking motives. Moreover, higher-level hospitals, based on their upstream position within the medical system, have monopolized some of the information on resources in the pharmaceutical market, allowing them to increase their bargaining power and to actively raise the price of medicine, causing the overall price of medicine to be generally higher [45]. In addition, information asymmetry between doctors and patients is also an important reason for the increase in medical costs. Doctors do not know much about their patients, which can easily lead to defensive medicine and overmedication, and patients blindly choose large hospitals for treatment due to their nervousness about their conditions, exacerbating healthcare costs. Ultimately, medicine prices and sales are both too high, resulting in patients paying more in reality than they expected to pay. This increased economic burden has also led to the deterioration of doctor–patient relationships [46].

A hierarchical medical treatment policy can reduce the cost of medical care for residents by promoting vertical integration and synergy among medical institutions. First, the hierarchical medical treatment policy saves patients’ time and energy costs by improving the rational allocation of medical resources between primary care institutions and specialized hospitals so that patients can seek medical treatment close to their homes, avoiding the situation where they are forced to queue up in large hospitals for registration because of less serious conditions [47]. At the same time, the hierarchical medical treatment policy encourages beneficiaries to seek medical treatment in accordance with the principle of hierarchical access through the first consultation of primary health care institutions and hierarchical referral, which effectively reduces medical accidents and medical risks due to inappropriate medical treatment and reduces the uncertainty of medical costs. Second, a hierarchical medical treatment policy is meant to improve medicine and medical management systems to save medical costs [29]. Regarding expenditure reductions, the policy has improved the existing medical and medical management systems that are still insufficient, including through the implementation of a zero-mark-up reform for the price of drugs and a reform of the doctor salary management system, and has prohibited doctors from obtaining revenue from drugs to eliminate profit-seeking practices such as prescribing excess medicines and providing excessive medical treatments. In addition, the supply side of the pharmaceutical market has been standardized, the bargaining power of medical institutions has been limited, and reasonable medicine prices have been restored [41]. In this way, the cost of medical treatment can be controlled, and the financial burden on patients can be reduced. Finally, hierarchical medical treatment policies also save medical risks and medical costs by promoting good interactions between doctors and patients. The close contact between primary care providers and patients enables doctors to better understand patients’ conditions and needs, to carry out diagnosis and treatment in a more timely and accurate manner and to reduce unnecessary medical checkups and treatments, which in turn saves healthcare expenditures [9].

Hence, we propose the following specific hypotheses:

##### Hypothesis 1

The implementation of the hierarchical medical treatment system can effectively improve doctor–patient relationships.

##### Hypothesis 2

The hierarchical medical system may improve doctor–patient relationships by the optimization of resource allocation.

##### Hypothesis 3

The hierarchical medical system may improve doctor–patient relationships by the saving of medical cost.

## Methods

### Model setting and variable definitions

As an administrative intervention, hierarchical medical treatment policy can be regarded as an exogenous variable. Since medical relations are affected by multiple factors, to determine the “policy treatment effect” of hierarchical medical treatment, the time-varying difference-in-differences method, which is a common policy evaluation method, was used to reflect the policy effect by comparing the differences in doctor-patient relations between the experimental group and the control group before and after the implementation of the hierarchical medical treatment policy. Since 2015, China has launched two batches of pilot cities for hierarchical medical treatment policy. The first pilot project was launched in 2015 and included 270 cities. The second pilot batch was identified in 2017 and included 51 cities. On the one hand, the implementation of the pilot policy may have made a difference before and after the implementation of the policy; on the other hand, it may also have made the doctor-patient relationship different between the pilot cities and the nonpilot cities at the same time point. Model regression estimation based on this dual difference can effectively control the influence of other syntemporal policies and the prior differences between pilot cities and nonpilot cities and then identify the net impact of policy on the doctor-patient relationship. Therefore, the time lag in the implementation of hierarchical medical treatment policy in different regions of China creates a favorable “quasinatural experiment” situation for evaluating its effects. Therefore, this paper uses a time-varying difference-in-differences model for evaluation. Pilot cities were used as the treatment group, and nonpilot cities were used as the control group to assess the effect of the pilot policy of hierarchical medical treatment. The specific form is shown in formula (1):

1 $${Relationship}_{it}={\alpha }_{1}+{\delta }_{1}{HMS}_{it}+{\theta }_{1}{control}_{it}+{\phi }_{i}+{\gamma }_{t}+{\epsilon }_{it}$$

In formula (1), ${Relationship}_{it}$is the explained variable, that is, the quality of doctor–patient relationships. ${HMS}_{it}$ is the core explanatory variable, a dummy variable that equals one for the years after the hierarchical medical treatment policy was implemented and zero otherwise. ${\delta }_{1}$is the key parameter of interest; it measures the pre–post change in the quality of doctor–patient relationships, thereby indicating the effect of the policy on doctor–patient relationships. ${control}_{it}$ represents the set of all control variables, and ${\theta }_{1}$ is its coefficient. ${\phi }_{i}$ represents the city fixed effects, ${\gamma }_{t}$ represents the year fixed effects, and ${\epsilon }_{it}$ is the error term. The subscripts $i$ and $t$ indicate the index cities and years, respectively.

In addition to the hierarchical medical treatment policy, there are many exogenous factors that affect doctor–patient relationships. Therefore, following Liang et al. [48] and Zhang et al. [15], the following control variables are used in this article: The economic development level of the prefectural-level cities is captured by per capita GDP ($gpc$). The carrying capacity of medical institutions is captured by the number of medical practitioners (and assistants) per 10,000 people ($pnob$) and the number of hospital beds per 10,000 people ($pnod$). The level of medical insurance coverage is captured by the number of insured individuals per 10,000 residents ($pmi$).

### Sample selection and data sources

The study period used in this paper is from 2012 to 2019. This period is chosen because the hierarchical medical treatment policy was officially implemented at the national level in 2015, and the dynamics of the policy implementation effect can be better observed over an 8-year period. Considering the implementation time and lag of the pilot policy, as well as the operability of this study, this paper determines the starting time of the two batches of hierarchical medical treatment pilot cities as 2015 and 2017. At the same time, this paper regards all the counties and districts in the pilot cities of hierarchical medical treatment as pilot areas of hierarchical medical treatment. In addition, if a city has multiple policy implementations, it is defined as the earliest time.

Most of the existing studies use the form of questionnaire to obtain the relevant data of doctor-patient relationship through indicators such as patients’ trust or patient satisfaction. However, the above indicators are due to their strong subjectivity and the relatively small coverage of the questionnaire, which can easily lead to the distortion of causal inference. Therefore, this paper draws on the studies of Mao et al. [20] and Liu et al. [19], and uses the mean of daily online search frequency to represent the level of doctor-patient relationshipin the city (district). Among them, the daily search keywords of people in each city are medical disturbance, medical accidents, medical disputes, medical injuries and doctor-patient conflict. The daily online search information mainly comes from the Baidu search index [49]. The Baidu index is based on the number of searches of internet users and is generally weighted to average by the number of keywords appearing in the network, thus reducing the interference of other factors. At present, the index has been widely used in existing research and can more objectively measure the doctor-patient relationship [50, 51].

In the mechanism analysis, the effect of optimization the allocation of resources and the effect of medical cost reductions are also included. The core driver of patients’ decisions to obtain their healthcare services in a primary care facility is the improvement of the quality of medical services rather than a reduction in economic costs. Therefore, an increase in the rate of outpatient visits to primary care facilities ($pvr$) indicates that patients have been successfully guided downward. That is, the quality of services at primary care facilities has improved effectively due to the optimization of the allocation of resources^24^. A reduction in the cost of medical treatment for residents ($rmc$) means that the price of medicine has been controlled, the additional expense of repeated medical services can be avoided, and the level of medical insurance reimbursement has increased. The percentage of outpatient visits to primary care facilities ($pvr$) is calculated as the number of outpatient visits to primary care facilities divided by the number of outpatient visits to all medical institutions. Residents’ medical care costs are captured by per capita health care expenditures ($rhe$), and regional prices are used to adjust expenditures to reduce the impact of differences in prices on expenditure indicators.

Second, the original data of the mechanism variables and control variables used in this article come from the China Health Statistics Yearbook, the statistical yearbooks of the different provinces, and the statistical yearbooks of the prefectural-level cities. For this article, the data were cleaned before use, and city observations for which data were missing for many years were dropped. For some observations for which data were missing for only some years, the missing values were estimated via interpolation. In addition, to avoid interference from outliers, all variables were winsorized at the 1st and 99th percentiles, and to reduce heteroscedasticity, some continuous variables were logarithmically transformed. Eventually, data on 286 prefectural-level cities were obtained. The descriptive statistics for the variables are shown in Table 1.

**Table 1:** Variable definitions and descriptive statistics

Variable	Description	Observations	Mean	SD
*sfd*	*Average daily frequency for internet searches of the term “doctor–patient conflicts” and related keywords*	2187	10.521	0.205
Ln(*gpc*)	*Logarithm of per capita GDP*	2229	10.705	0.553
Ln(*pnob*)	*Logarithm of hospital beds per 10,000 people*	2146	3.05	0.96
Ln(*pnod*)	*Logarithm of medical practitioners (and assistant) per 10,000 people*	2137	2.321	0.969
Ln(*pmi*)	*Logarithm of the number of insured individuals per 10,000 residents*	1771	5.154	0.936
Ln(*rhe*)	*Logarithm of per capita health care expenditure*	1824	6.853	0.385
*pvr*	*Share of visits to primary care facilities*	1275	0.4868	0.133

## Baseline regression analysis

### Baseline regression

First, Model (1), which does not include any control variables, estimates the impact of the hierarchical medical treatment policy on doctor–patient relationships. The results are shown in columns (1) of Table 2. The results show that the policy has a significant negative effect on the frequency with on the average daily frequency of internet searches for similar keywords. These estimates are negatively significant at the 1% levels, which means that the policy has significantly improved doctor–patient relationships. However, the above results do not exclude the influence of other factors. To identify the net effect of the policy, columns (2) show the impact of the hierarchical medical treatment policy on doctor–patient relationships more accurately after accounting for other factors. After adding control variables, the policy is found to have a significant negative effect on the average daily frequency of internet searches for related keywords. The findings of this research are stable. Hypothesis 1 is confirmed.

Regarding the control variables, the estimated coefficient on per capita GDP is significantly negative. This shows that the rising level of economic development has increased the affordability of medical expenses, which has relieved the strain on doctor–patient relationships. The estimated coefficients for the number of hospital beds per 10,000 people and the number of medical practitioners (and assistants) per 10,000 people are both significantly negative, indicating that the increase in the number of supply-side medical resources has eased the difficulty of receiving medical services and has optimized those services. The estimated coefficient of the number of insured individuals per 10,000 residents is significantly negative, indicating that on the demand side, reimbursement guarantees from medical insurance reduce the high cost of receiving medical services and improve doctor–patient relationships to an extent.

**Table 2:** Baseline regression results

	(1)	(2)
*HMS*	-0.615*** (-0.145)	-0.523** (-0.257)
*Lngpc*		-0.972* (-0.545)
*Lnpnob*		-0.281** (-0.135)
*Lnpnod*		-4.152* (-2.341)
*Lnpmi*		-0.013** (-0.006)
*N*	2187	1689
*R* ^*2*^	0.285	0.295

*Notes.* (1) * indicates significance at the 10% level; ** indicates significance at the 5% level; and *** indicates significance at the 1% level. (2) Robust standard errors clustered at the city level are reported in parentheses. (3) The constant term and the estimated year and city fixed effects are not reported in the table. These notes also apply to the following table

### Common trends test and dynamic impact analysis

The key premise of the time-varying difference-in-differences model is the parallel trend assumption, that is, testing whether the pilot cities of hierarchical medical treatment and nonpilot cities have the same change trend before the policy impact. This paper uses dynamic effect analysis to show the dynamic impact of the hierarchical medical treatment pilot policy on the doctor-patient relationship [52, 53]. The analysis in this part of the paper is based on formula (2).

2 $${Relationship}_{it}=\alpha +\sum _{t=-4}^{4}\delta {HMS}_{i}\times {Trend}_{t}+\theta {control}_{it}+{\phi }_{i}+{\gamma }_{t}+{\epsilon }_{it}$$

In the formula, ${HMS}_{i}\times {Trend}_{t}$ represents the interaction between the city dummy variable and the time trend dummy variable for the hierarchical medical treatment policy. $\rho$ is its estimated coefficient, and the range of $t$ is $-4\le t\le 4$. If the value of $t$ equals three, then the time trend dummy variable equals one for observations in the first three years of the implementation of the hierarchical medical treatment policy and zero otherwise. If none of the estimated coefficients $\delta$ are significant, the common trend assumption is satisfied. In addition, interaction terms between the city dummy variables and the dummy variables of the event study after the implementation of the hierarchical medical treatment policy are incorporated into the regression model to test whether there is a lag in the effect of the policy.

**Fig. 2:**
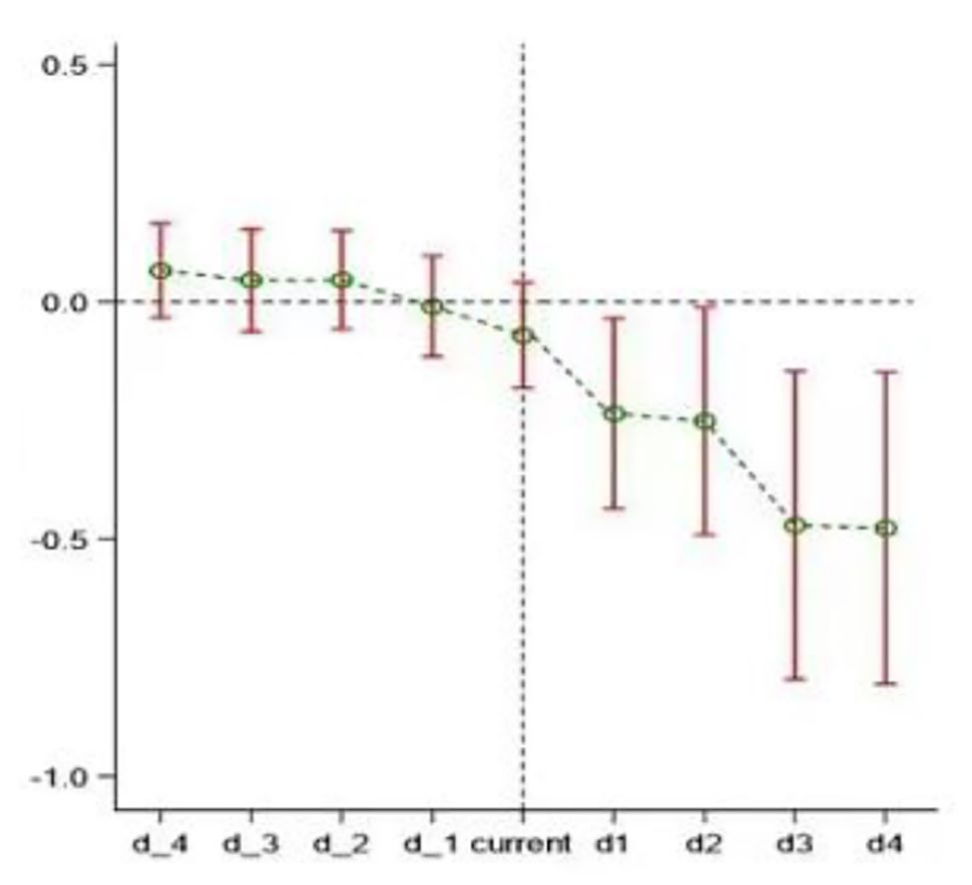
Parallel trend test

The results of the parallel trend test are shown in Fig. 2. None of the regression coefficients before the implementation of the hierarchical medical treatment policy are significant, indicating that there is no significant difference in the level of doctor-patient relationships between the pilot cities and the nonpilot cities and that there is no significant difference between the doctor-patient relationships between the pilot cities and the nonpilot cities. After the implementation of the hierarchical medical treatment policy, the doctor-patient relationships between the pilot cities and nonpilot cities are significantly different; i.e., the sample satisfies the parallel trend test. In addition, Fig. 2 also shows that the effect of the tiered diagnosis and treatment policy was not obvious in the year of implementation and began to show a significant improvement after one year of implementation. This improvement effect continued to increase over time, suggesting that the tiered diagnosis and treatment policy has produced a sustained improvement effect on the doctor-patient relationship. This result reinforces the credibility of the baseline regression results in this paper.

### Robustness test

To ensure the robustness of the baseline regression, a series of robustness tests are conducted in this article. First, placebo tests are conducted. Then, the influence of other confounding policies is eliminated, an alternative measure of the quality of doctor-patient relationships is used, and reverse causality is eliminated.

#### Placebo test

According to the regression results in Table 2, the hierarchical medical treatment policy significantly improved doctor-patient relationships. However, some random factors may have been included in the regression, resulting in statistically significant indicators. Therefore, to eliminate interference from other characteristics of the pilot cities in the baseline regression results, a placebo test is used to determine whether the improvements in the doctor-patient relationship are caused by other random factors and whether the impact is real or objective. First, the data are divided into groups by city, and then a treatment group is randomly generated. This process is repeated 1000 times, and then the groups are used to estimate 1000 regressions. From these regressions, the distribution of the counterfactual coefficient ${\alpha }^{counterfactual}$ is obtained. If the hierarchical medical treatment policy does not improve doctor-patient relationships, then ${\alpha }_{1}$ should be in the middle of the ${\alpha }^{counterfactual}$ distribution.

**Fig. 3:**
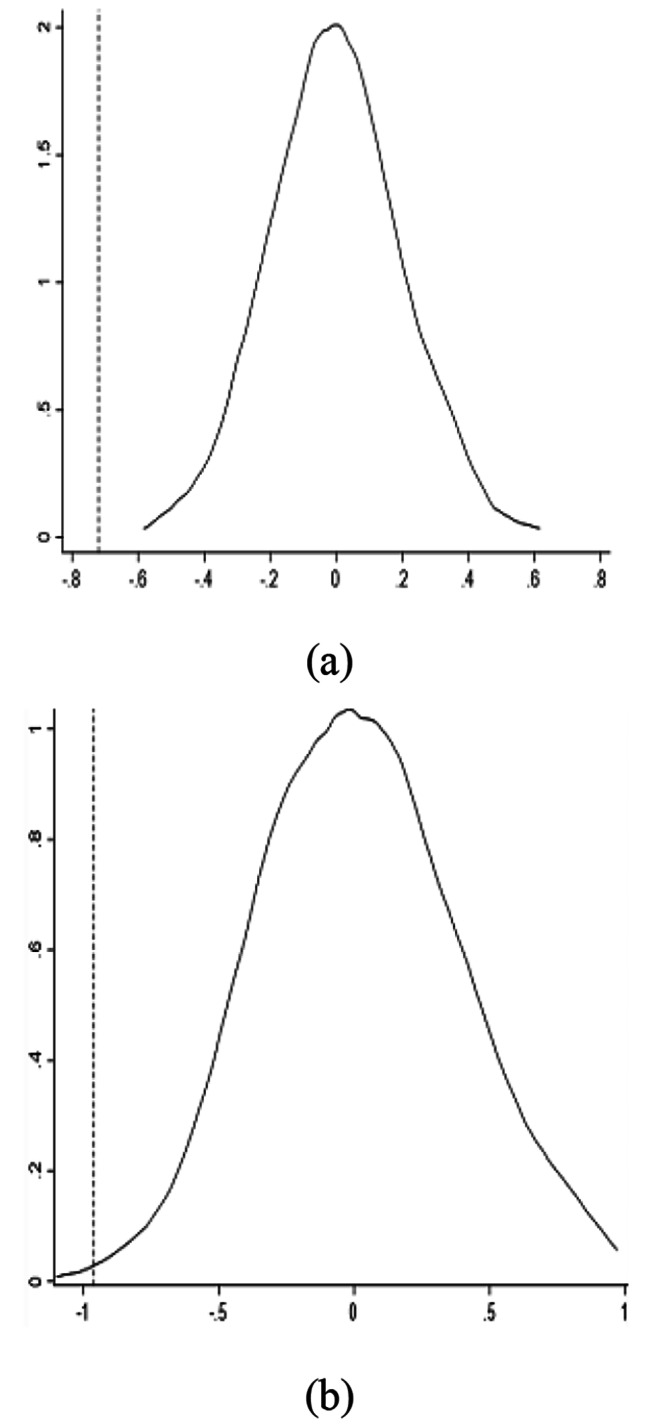
Placebo test (1000 random simulations)

As shown in Fig. 3, the dashed line is the true value of ${\alpha }_{1}$ obtained from formula (1), and the solid line is the distribution of ${\alpha }^{counterfactual}$ from 1000 random simulations. As shown in Fig. 3(a), the true value of the regression coefficient ${\alpha }_{1}$ on the frequency with which the terms “doctor-patient conflicts” and other keywords are included in local newspapers is significantly smaller than the counterfactual simulation coefficient ${\alpha }^{counterfactual}$. According to Fig. 3(b), the true value of the regression coefficient ${\alpha }_{1}$ on the frequency of average daily internet searches for “doctor–patient conflicts” and related keywords is also significantly smaller than that of the counterfactual coefficient ${\alpha }^{counterfactual}$. Therefore, the placebo test results show that the baseline regression results are robust.

#### Elimination of the influence of other confounding policies

A critical illness insurance policy was also implemented during the study period, along with the hierarchical medical treatment policy. In August 2012, six ministries and commissions of the General Office of the State Council of the People’s Republic of China issued the “Guiding Opinions on Implementing the Critical Illness Insurance Policy for Urban and Rural Residents”, a pilot for the critical illness insurance program was launched. As of December 2015, the critical illness insurance program had been implemented in all 31 provinces and province-level municipalities across the country, and there was a high degree of overlap in the pilot cities of that policy and of the policy studied in this article. Therefore, the critical illness insurance policy may confound the baseline evaluation of the effect of the hierarchical medical treatment policy on doctor–patient relationships. To eliminate the interference of the above policy in the results from the baseline regression, the following equation is established on the basis of formula (1):

3 $${Relationship}_{it}={\alpha }_{1}+{\delta }_{1}{HMS}_{it}+{\sigma }_{1}{did01}_{it}+{\theta }_{1}{control}_{it}+{\phi }_{i}+{\gamma }_{t}+{\epsilon }_{it}$$

In formula (3), ${did01}_{it}$ is the difference-in-differences estimator of the critical illness insurance policy. If city $i$ implements the critical illness insurance policy in year $t$, then ${did01}_{it}=1$ for city *i* in year $t$ and in subsequent years; otherwise, it equals zero. The corresponding estimation results are reported in columns (1) of Table 3. After controlling for the critical illness insurance policy, the hierarchical medical treatment policy still significantly improved doctor–patient relationships, which indicates that the estimated results in this article are robust.

#### Alternative measures for doctor–patient relationships

The number of court cases with keywords such as “doctor–patient conflicts” at all levels in each city is a direct manifestation of the quality of doctor–patient relationships at the legal level. Following Xiao et al. [12] and Jiang et al. [2], the number of such court cases at all levels in each city ($cod$) is used to replace $sfd$ in Table 2 as the explained variable in the regressions. The results in column (2) of Table 3 show that the estimated coefficient is significantly negative at the 10% level, which means that after the implementation of the hierarchical medical treatment policy, the number of court cases with keywords such as “doctor–patient conflicts” at all levels in each city decreased significantly; that is, doctor–patient relationships improved significantly.

**Table 3:** Estimated robustness test results

	(1)	(2)	(3)
	*sfd*	*cod*	*sfd*
*HMS*	-0.512* (-0.295)	-0.328* (-0.189)	-0.520* (-0.276)
*did01*	-1.161** (-0.489)		
*Controls*_*t−1*_			Yes
*Control variables*	Yes	Yes	
*N*	1689	1689	1689
*R* ^*2*^	0.276	0.409	0.314

*Notes*. The data on the number of cases with keywords such as doctor-patient disputes at all levels come from the judgment document network: https://wenshu.court.gov.cn/

#### Elimination of reverse causality

In the baseline regression, there may be reverse causality in the relationship between the control variables and the explained variable. For example, the more medical practitioners (and assistants) per 10,000 people there are, the more accessible healthcare services are for patients, which in turn helps improve doctor–patient relationships. However, as the relationship between doctors and patients deteriorates, the number of students applying to medical majors on college entrance examinations is negatively affected, reducing the number and quality of students pursuing the medical profession, which is expected to have a long-term adverse effect on the supply of medical talent. Ultimately, this would affect the number of medical practitioners (and assistants) per 10,000 people. Therefore, to eliminate this reverse causality, the one-period lag of each control variable is used. The results are shown in columns (3) of Table 3. The empirical results for the main variables show no significant change.

In summary, whether a placebo test is conducted, the influence of other confounding policies is eliminated, an alternative measure of the quality of doctor–patient relationships is used, or the effect of reverse causality is eliminated, the results show that the hierarchical medical treatment policy had a significantly positive effect on doctor–patient relationships.

### Solutions to endogeneity

The government’s selection of pilot cities for the hierarchical medical treatment policy was not completely random, which means that the impact of the implementation of the policy on doctor–patient relationships may still be biased by potential endogeneity; therefore, in this study, the instrumental variable method was adopted to resolve endogeneity. The number of medical institutions in each city in 1995 is used to construct the instrumental variable for the hierarchical medical treatment policy. The number of medical institutions in each city in 1995 reflects the status of the medical infrastructure in each city in that year. Due to the path dependency in medical infrastructure, cities that have developed relatively complete medical infrastructure early on often have higher-quality medical institutions and more standardized medical procedures. Such cities better reflect the context of the reform and the effect of the experiment, and therefore, they have the great advantage of being selected as pilot cities for hierarchical medical treatment policy. Therefore, the instrument satisfies the relevancy condition. Moreover, it is difficult to believe that there is a correlation between the number of medical institutions in the past and current doctor–patient relationships; therefore, the instrument satisfies the exogeneity condition. Since the number of medical institutions in each city in 1995 was cross-sectional only, it cannot be directly used for estimation in the panel model. Following Sun et al. [54], the interaction terms between the logarithm of the number of medical institutions in each city in 1995 ($miec$) and the time dummy variable ($post$) are used as instrumental variables. The estimation results for the first and second stages are shown in Tables 4 and 5.

Table 4 shows that the effect of $miec\times post$ on the implementation of the hierarchical medical treatment policy is significantly positive, indicating that there is a strong correlation between the number of medical institutions and the implementation of the hierarchical medical treatment policy.

**Table 4:** First-stage estimation results for the instrumental variable analysis

	HMS		HMS
*miec×post*_*2012*_	0.023***	*miec×post*_*2016*_	0.033***
	(0.002)		(0.002)
*miec×post*_*2013*_	0.021***	*miec×post*_*2017*_	0.036***
	(0.003)		(0.002)
*miec×post*_*2014*_	0.021***	*miec×post*_*2018*_	0.038***
	(0.004)		(0.002)
*miec×post*_*2015*_	0.028***	*miec×post*_*2019*_	0.026***
	(0.005)		(0.001)
*Control variables*	*Yes*		
*N*	1689		

Table 5 shows that the Kleibergen–Paap rk Wald F statistic for the Stock–Yogo weak identification test is greater than the relevant critical value at the 10% level; this result implies that weak instrumental variables are not a problem. The value corresponding to the Kleibergen–Paap rk LM statistic $\rho$ is less than 0.01, which implies that the model is not underidentified. The Hansen J statistic is insignificant, which demonstrates that the instrumental variable model is also not overidentified. The hierarchical medical treatment policy has a significant negative impact on the average daily frequency of internet searches for related keywords. This shows that after addressing the endogeneity issue, hierarchical medical treatment policy is still found to improve the relationship between doctors and patients.

**Table 5:** Second-stage estimation results for the instrumental variable analysis

	2SLS
*HMS*	-0.518**
	(-0.211)
*Control variables*	*Yes*
*N*	1424
*Kleibergen–Paap rk LM Statistic*	44.362***
	(0.000)
*Kleibergen–Paap rk Wald F Statistic*	61.714
	(18.261)
*Hansen J Statistic*	9.372
	(0.880)

*Notes*. The values in parentheses for the Kleibergen–Paap rk LM statistic and Hansen J statistic are *P* values, and the values in parentheses for the Kleibergen–Paap rk Wald F statistic are the critical values for the Stock–Yogo weak identification test at the 10% level

## Mechanism analysis

According to the benchmark regression, the pilot policy of hierarchical medical treatment effectively improved the doctor-patient relationship. According to our theoretical analysis, through the optimization of resource allocation and medical cost savings, the pilot-tier diagnosis and treatment policy effectively guides patients to primary health institutions so that patients can obtain more comfortable and caring services after triage and reduces the opportunity cost of patients’ downward consultation, thus improving the trust between doctors and patients and their satisfaction with consultation and ultimately improving the doctor-patient relationship. To further empirically test the mediating effect of the tiered diagnosis and treatment policy on the doctor-patient relationship by the optimization of resource allocation and saving of medical cost, this paper draws on the research of Jiang [55] to construct model (4), and the specific results are shown in Table 6.

4 $${Mediation}_{it}={\alpha }_{1}+{\delta }_{1}{HMS}_{it}+{\sigma }_{1}{did01}_{it}+{\theta }_{1}{control}_{it}+{\phi }_{i}+{\gamma }_{t}+{\epsilon }_{it}$$

Where ${Mediation}_{it}$ are *pvr and rhe*, respectively. The other variables are consistent with the previous meaning and are not repeated.

**Table 6:** Estimation results for the mechanism analysis

	(1)	(2)
	*pvr*	*rhe*
*HMS*	0.085* (0.049)	-0.094** (-0.041)
*Control variables*	Yes	Yes
*N*	1275	1689
*R* ^*2*^	0.547	0.662

The results are shown in Table 6. Column (1) shows that the estimated coefficient representing the effect of the hierarchical medical treatment policy on the rate of outpatient visits to primary care facilities is 0.085, which is significant at the 10% level, indicating that the implementation of the tiered diagnosis and treatment policy can optimize the allocation of medical resources at all levels and improve the quality of primary healthcare services through “diversion”, thus successfully guiding residents to seek downward consultation and increasing the rate of primary consultation for residents. The rate of primary care consultation for residents has increased. An increase in the rate of residents’ primary care will not only alleviate the phenomenon of doctors receiving more than 70% of “regular, general, and multiple” patients who are in a state of overload for a long period [41] but also prevent patients’ satisfaction with medical care from being affected by the long waiting time for medical care and the effect of diagnosis and treatment being poorer than expected [36], which can effectively reduce the contradiction between doctors and patients and build an important way of good doctor-patient relationships. The hierarchical medical treatment policy can improve the doctor-patient relationship through the optimization of resource allocation and subsequently improve the doctor-patient relationship.

Column (2) shows that the estimated coefficient of the hierarchical medical treatment policy on per capita healthcare expenditure is negative and significant at the 5% level, indicating that the implementation of the hierarchical medical treatment policy reduces per capita healthcare expenditure and effectively saves residents’ visits to clinics. According to the theoretical analysis section, scholars such as Yip et al. [29] and Chu et al. [9] have pointed out that, due to the phenomena of “medication for medicine”, “excessive medical care”, “defensive medicine” and other phenomena, the cost of medical treatment for residents has gradually increased, and the actual payment is higher than the expected payment level, which in turn leads to low patient satisfaction with medical treatment and tensions between doctors and patients. The hierarchical medical treatment policy can improve the doctor-patient relationship through medical cost savings.

In summary, the optimization of resource allocation and medical cost savings are the significant paths through which hierarchical medical treatment policies improve doctor-patient relationships, and hypotheses 2 and 3 are proven.

## Heterogeneity

### Extent of aging within the urban population

The data are taken from the seventh National Chinese census, according to which the sample is divided into nonaging cities ($nar$), aging cities ($lar$) and severely aging cities ($har$) depending on the extent of aging. If a given city’s population of individuals aged 65 years or over accounts for more than 14% of the total population, it is considered a severely aging city ($har$). If the population aged 65 or older is more than 7% but less than 14%, it is an aging city ($lar$). Finally, if that population is less than 7% of the total population, it is considered a nonaging city ($nar$). In addition, a permutation test is used to verify the differences between the city groups. Table 7a shows that the empirical *p* value from the permutation test regarding the difference between groups in the extent of aging within the relevant city populations is less than 0.1, indicating the existence of heterogeneous aging at the city level. The reason may be that compared with young people, elderly individuals are relatively more likely to suffer from illnesses or to live with diseases, and the quality of medical care they need is more stringent; thus, the possibility of going to a higher-level hospital will also increase. In addition, elderly people’s long-term practice of going to the hospital when sick makes it difficult to change their health-seeking behavior due to these existing habits [56]. Therefore, it is more difficult for hierarchical medical treatment policies to guide elderly patients to visit primary care facilities.

The regression is based on three groups of cities with different degrees of population aging, and the results are shown in Table 7a. The estimated coefficients of hierarchical medical treatment policy are all significantly negative, indicating that the policy significantly improved the relationship between doctors and patients in all sample cities regardless of the degree of population aging. The results also show that the absolute value of the estimated coefficients on the hierarchical medical treatment policy in columns (1) to (3) gradually decreases, indicating that as the extent of population aging intensifies, the marginal effect of the policy on doctor–patient relationships fades.

**Table 7a:** Estimation results for heterogeneity analysis 1

	(1) nar	(2) lar	(3) har
*HMS*	-0.526* (-0.294)	-0.523* (-0.295)	-0.522* (-0.279)
*control variables*	Yes	Yes	Yes
*permutation test*	(1)vs. (2) 0.037*** (0.011)	(2)vs. (3) 0.048** (0.019)	(1)vs. (3) 0.074*** (0.003)
*N*	168	1128	992
*R* ^*2*^	0.334	0.321	0.323

### City administrative level

Cities at different administrative levels in China have different development levels, resource endowments and development histories. Municipalities, provincial capitals and subprovincial cities are defined as focal cities, and general prefectural-level cities are defined as general cities. As the centers of development within a region, focal cities have large amounts of scientific and educational resources, which are conducive to training excellent medical students and forming a sufficiently large reserve of medical talent within the city. Moreover, their infrastructure is complete, which means that they often siphon talent and resources away from other areas. Therefore, when the hierarchical medical treatment policy was implemented, the quality of primary medical services in focal cities could be maximized, thereby effectively improving doctor–patient relationships. However, the supporting facilities in general cities lag behind those of focal cities, which makes such cities less attractive to medical workers than focal cities. Therefore, cities of different administrative levels may have benefited differently from the implementation of the policy.

This study defines a dummy variable for whether the observed city is a focal city ($hcl$). This variable equals one if the observed city is a focal city and zero otherwise. In addition, an interaction item between this dummy variable and the policy variable is included in the estimation. The results are shown in column (1) of Table 7b. The estimated coefficient on the interaction between the dummy variable indicating focal cities and the hierarchical medical treatment policy is significantly negative at the 5% level, indicating that the hierarchical medical treatment policy affects the relationship between doctors and patients in focal cities. This positive effect is stronger in focal cities than in general cities.

**Table 7b:** Estimation results for heterogeneity analysis 2

	(1)	(2)
	*Focal city*	*High level of fiscal spending on health care*
hcl*HMS	-1.925^**^ (-0.832)	
hfhe*HMS		-3.053^**^ (-1.363)
*Control variables*	Yes	Yes
*N*	1689	1689
*R* ^*2*^	0.271	0.285

### City-level government expenditures on health care

The government has always been a powerful driving force in the development of Chinese society at all levels [57]. Differences in the level of government financial support for medical care directly affect the development level of primary care facilities. In accordance with the practices of the previous literature, fiscal spending on health care ($fhe$) is used to measure the government’s role in the development of medical and health services. A high level of fiscal spending on health care increases the medical resources available to primary care facilities, thereby improving the level of treatment and increasing the carrying capacity of those facilities. A lower level of government expenditure on health care may slow its development. Therefore, the effect of the hierarchical medical treatment policy on primary care facilities, its main target, may vary across cities according to the level of fiscal spending on health care.

This study defines a dummy variable for whether a given city has a high level of fiscal spending on health care” ($hfhe$), which equals one if health care spending in the observed city is higher than the median and zero otherwise. In addition, an interaction term between the dummy variable for high levels of fiscal spending on health care and the hierarchical medical treatment policy is included in the estimation. The results are shown in column (2) of Table 7b. The estimated coefficient on the interaction term between the fiscal spending dummy variable and the hierarchical medical treatment policy is significantly negative at the 5% level, indicating that the positive effect of the policy on the relationship between doctors and patients is stronger in cities with high levels of fiscal spending on health care than in cities with low levels of fiscal spending on health care.

## Conclusions and implications

To solve the existing problem of the irrational allocation of medical resources in China, hierarchical medical treatment reform, which includes a series of subreform policies, has been implemented in most cities in China, and it has had an important impact on building a harmonious society. In this paper, to explore the effect of hierarchical medical treatment policy on resolving the problem of “difficult and expensive to see the doctor”, based on panel data on prefectural-level cities in China from 2012 to 2019, a dynamic difference-in-differences model was constructed to evaluate the impact of the quasinatural experiment of the hierarchical medical treatment policy on doctor–patient relationships. The findings can be summarized as follows: First, we found that the expansion of the hierarchical medical treatment policy significantly improved doctor–patient relationships. This finding is robust to a set of robustness and endogeneity tests. Second, improving doctor-patient relationships can be indirectly realized by the optimization of resource allocation and saving of medical costs. Third, this article also studies the effect of policies on cities with different levels of aging within their population, cities at different administrative levels, and cities with different levels of fiscal spending on health care. The results show that the marginal effect of the pilot policy on doctor–patient relationships decreases with an increase in the degree of aging within the population. The pilot policy also has a stronger positive effect on doctor–patient relationships in focal cities and in cities with high levels of fiscal spending on health care.

Building harmonious doctor–patient relationships requires a focus on ensuring the stability and endurance of positive interactions between doctors and patients. In this article, the hierarchical medical treatment policy is found to play a crucial role in ensuring the long-term healthy development of doctor–patient relationships. Therefore, it is essential to further deepen the implementation of hierarchical medical treatment policy and to adopt the following measures to increase the effect of the optimization of the allocation of resources and reductions in medical costs, thus maximizing the positive effect of the policy on the quality of doctor–patient relationships.

First, the pilot program should be continuously expanded to deepen the reform of graded diagnosis and treatment. On the one hand, local governments at all levels should adhere to practical approaches, adapt to local conditions, and promote the pilot work of graded diagnosis and treatment in a variety of forms. Drawing on the successful experience of the existing tiered diagnosis and treatment model, medical institutions can continue to carry out tiered diagnosis and treatment by uniting medical institutions at different levels, implementing differentiated payments for medical insurance, setting up multidisciplinary teams of medical specialists, and perfecting norms for the diagnosis and treatment of common diseases and the use of medication. On the other hand, we will further explore the reform measures of graded diagnosis and treatment, balance the interests of medical institutions at different levels, and create a mechanism for sharing medical information, medical resources, and medical supervision among medical institutions at different levels to promote the healthy development of the primary healthcare system.

Second, we are guiding the sinking of high-quality medical resources and upgrading the capacity of primary medical and health-care services. On the one hand, we will continue to create an environment in which primary health care personnel can “stay”. The attractiveness of primary health-care institutions to outstanding medical personnel will continue to be enhanced by means of improved remuneration and optimized career prospects. At the same time, a long-term talent training mechanism has been established for direct-transfer primary health care organizations, emphasizing vocational skills training and retraining the medical service capabilities of existing personnel. On the other hand, the infrastructure of primary healthcare organizations should be further improved. In accordance with the positioning and responsibilities of primary medical and health-care institutions under the hierarchical medical treatment system, the government should increase financial support and continue to provide the necessary support for the construction and development of primary medical and health-care institutions.

Third, medical costs are saved, and the primary medical and health-care service system is improved. Promoting the in-depth integration of “Internet + medical services” will save residents’ medical costs. Promote electronic medical records, wisdom services, wisdom management, and “trinity” construction; comply with the development trend of remote medical and internet diagnosis by means of “Internet +” in combination with online consultation, offline diagnosis of medical new situations; provide patients with low-cost, highly convenient medical information access channels; save the indirect cost of patients, reduce patients’ medical expenses, and improve information technology services on the basis of stock resource intersection increments to improve patients’ medical experience.

Fourth, city differentiation policies should be established to fully address the effects of pilot-tiered diagnosis and treatment policies in disadvantaged cities. On the one hand, attention should be given to the medical service needs of middle-aged and elderly people in cities with aging populations, and outpatient clinics for elderly patients should be opened in primary care facilities. Digital medical equipment and apps for healthcare services that are convenient for elderly people seeking medical treatment should be developed to help elderly people cross the “digital divide” in medical services. Elderly individuals should be guided to seek medical treatment at lower-level institutions, which would reduce their medical costs. On the other hand, the urbanization of general cities should accelerate, basic facilities that support urban life should be constructed, and public transportation infrastructure should be built to improve the quality of urban life. In addition, a rural revitalization strategy should be implemented, mutually beneficial interactions between urbanization and rural revitalization should be strengthened, and government and medical resources should be sent to villages and local communities to stop focal cities from siphoning medical talent and resources away from such places and to promote the merger of urban and rural health institutions. Furthermore, central financial transfer payments to cities with low levels of fiscal spending on health care should be moderately increased to ease local financial pressures, thereby prompting those cities to increase the amount of their fiscal spending on health care and guiding them to support primary care facilities.

Although the conclusions of this paper are conducive to deepening the knowledge and understanding of the effects of hierarchical medical treatment policy, there are still some limitations. Future research should continue in the following directions: first, the doctor-patient relationship will have an impact on the allocation of medical resources, the structure of medical service supply, and the evaluation of the quality of medical services. In future research, we can explore the impact of the doctor-patient relationship on the above aspects to improve the effectiveness and efficiency of medical services and to provide new insights and ideas for new health care reform. Second, the hierarchical medical treatment policy will also have an impact on the demand for doctors and the supply side of medical services, and the impact of the hierarchical medical treatment policy on doctors and patients can be analyzed separately in future research to enhance the effect of the hierarchical medical treatment policy in a targeted manner. Third, The study of doctor-patient relationships can enrich and improve the theory of information asymmetry, doctor-patient trust theory, etc., and in the next step of the study, we can carry out theoretical research on doctor-patient relationships to enrich and expand health economics. Forth, countries worldwide have implemented a variety of health care policies to promote the construction of harmonious doctor-patient relationships, and in future research, we can further compare the health care policies of various countries to complement their strengths and weaknesses to develop a health care system that is suitable for their own national conditions and is effective.

## Data Availability

Free.
